# Effect of Laser Fluence and Scanning Strategy on Topography and Morphology of Femtosecond Laser-Structured Stainless-Steel Surfaces

**DOI:** 10.3390/mi17101189

**Published:** 2026-10-14

**Authors:** Robert Thomas, Timo Block, Hermann Seitz, Georg Schnell

**Affiliations:** 1 Microfluidics, Faculty of Mechanical Engineering and Marine Technology, University of Rostock, Justus-von-Liebig Weg 6, 18059 Rostock, Germany; timo7block@gmail.com (T.B.); hermann.seitz@uni-rostock.de (H.S.); georg.schnell@uni-rostock.de (G.S.); 2 Department Life, Light & Matter, Interdisciplinary Faculty, University of Rostock, Albert-Einstein-Str. 25, 18059 Rostock, Germany

**Keywords:** surface roughness, nano-/microstructures, surface texture, ablation threshold, laser pulse overlap, scanning line overlap, ultrashort laser pulse

## Abstract

Laser-induced, self-organized surface structures offer substantial functional advantages across a wide range of applications, including tribological, biomimetic, and medical applications. Among the key processing parameters governing the formation of laser-induced self-organized surface structures, laser fluence, pulse overlap (PO), and line overlap (LO) are particularly important. This study systematically investigates their influence on the morphology and topography of femtosecond laser-structured stainless-steel surfaces. For this purpose, the samples were irradiated with a 300 fs laser emitting linearly polarized light (λ = 1030 nm). Scanning electron microscopy revealed four surface morphologies: laser-induced periodic surface structures (LIPSS), micrometric ripples (MR), micro-crater structures, and pillared microstructures. Surface roughness measurements and morphological analyses revealed that an increasing PO promotes the transition from LSFL- and MR-dominated morphologies to crater and pillared microstructures at substantially lower fluences than increasing LO. The strong-ablation regime was therefore reached at lower fluences, resulting in earlier formation of complex hierarchical microstructures and a maximum surface roughness of Sa = 13.46 µm, compared with Sa = 6.90 µm obtained by varying LO. These findings demonstrate that PO has a stronger influence on local heat accumulation than LO and thus plays a more prominent role in governing the evolution of surface morphology and roughness.

## 1. Introduction

Over the past few decades, the micro- and nanostructuring of surfaces has become an increasingly important field within surface engineering and micromachining. Particularly in ultrashort-pulse laser technology, characterized by pulse durations in the femtosecond and picosecond range, the possibilities for surface processing have expanded significantly in terms of accuracy, resolution, and flexibility. Ultrashort-pulse laser technology enables highly precise material removal with minimal thermal damage to the unirradiated material [1,2,3]. Furthermore, high production efficiency can be achieved on a wide variety of materials by adjusting the applied laser energy and scanning speed [4,5]. These and other advantageous features have led, in recent years, to both a wide range of research work [4,5,6,7,8,9] and, consequently, numerous industrial applications [10,11,12,13]. In particular, interest has grown in the controlled modification of surface topography and morphology using ultrashort laser pulses [4,6]. On the one hand, the controlled modification of surface topography and morphology can be achieved through laser-inscribed structures and, on the other hand, through self-organized structures formed during single-beam processing. While laser-inscribed structures are generally limited to feature sizes comparable to the laser focus diameter, laser-induced self-organized structures can form with characteristic dimensions below the focused-beam diameter [4]. Sub-diffraction-limit features can also be written directly by advanced light-field modulation, such as longitudinal field enhancement [14]. However, this requires high-numerical-aperture focusing and serial writing, which currently limits its applicability to large-area surface processing. In particular, self-organized structures and their formation mechanisms have attracted significant scientific interest [15,16,17,18,19,20]. Their diverse applications range from improving tribological performance and reducing friction [10,21,22,23] to adjusting wettability and creating superhydrophobic surfaces [9,24,25,26,27] or creating biomedical and functional surfaces [23,28,29,30], and to optical and photonic functionalities [23,31,32], to name just a few.

Given the significant scientific interest in laser-induced, self-organized surface structures and their wide range of potential industrial applications, comprehensive parameter studies are needed to investigate the factors that influence their formation systematically. Existing research reveals that the topography and morphology of self-organized surface structures on metals are primarily determined by their respective formation mechanisms [4,5]. The formation mechanisms depend significantly on the applied laser fluence and scanning strategy [33,34]. Depending on the applied fluence, different ablation regimes can be classified. The first ablation regime is referred to as the gentle ablation regime. It starts with the gentle ablation fluence, which represents the minimum fluence required for ablation [35,36,37,38], and is characterized by low ablation rates governed by the optical penetration depth [4]. At higher fluences, the strong ablation threshold is exceeded, and the ablation depth is governed by the effective thermal penetration depth, resulting in considerably higher ablation rates (strong ablation regime) [4,37]. When the laser fluence is near or exceeds the gentle ablation fluence, laser-induced periodic surface structures (LIPSS, also known as “ripples”) begin to form [39]. There are essentially two types of LIPSS [5,35], distinguished by their spatial orientation and size. The so-called low-spatial-frequency LIPSS (LSFL) are typically oriented perpendicular to the polarization of the laser beam on metals. The spatial period (Λ_LSFL_) is equal to or slightly smaller than the irradiation wavelength [19,40]. So-called high-spatial-frequency LIPSS (HSFL) on metals are characterized by an orientation parallel to the polarization of the laser beam and typically exhibit a less pronounced surface topography compared to LSFL [4,40]. The spatial period Λ_HSFL_ is less than half the irradiation wavelength [19,40]. If the fluence is increased slightly compared to the gentle ablation fluence, the LIPSS with spatial period Λ_1_ are superimposed by micrometric ripples with spatial period Λ_2_, which are typically oriented perpendicular to the LIPSS orientation (parallel to the polarization of the laser beam) [36,41]. The mechanisms underlying the formation of micrometric ripples (MR) are still under investigation and debate. Various mechanisms have been considered so far, such as heat accumulation and absorption in the ripple valleys enhanced by electromagnetic effects or scattering, which leads to selective ablation and the formation of micrometric ripples [33,35,42].

It has been shown that even a slight increase in laser fluence beyond that required for micrometric ripples causes craters to form on surface inhomogeneities. According to Zuhlke et al. and Schnell et al., MRs serve as precursor cells for crater formation [35,42], while the craters themselves either develop within the MRs or arise from the re-deposition of material [35]. From these precursor cells, the inhomogeneities eventually grow further due to two mechanisms: preferential valley ablation (PVA) and heat-driven phenomena (vapor–liquid–solid (VLS) growth and melt flow) [35,42]. For the PVA mechanism, the growth of the crater is driven by light reflections on the side walls of the local cavity, which increases the local laser light fluence in the center of the crater [33,35,42]. For VLS and melt-flow-induced growth, the formation of self-organized structures is evoked by heat accumulation and the subsequent surface temperature offset, leading to material melting and redistribution [33,35,42]. Both mechanisms depend on the applied fluence and the laser scanning strategy. At relatively low fluences and pulse overlaps, PVA dominates; at higher fluences and pulse overlaps, VLS growth and melt flow dominate. Zuhlke et al. and Schnell et al. have shown that circular crater structures then form continuously covering the surface until self-organized microstructures are formed [35,42]. The resulting surface relief can be classified into two different types. Depending on the laser parameters used, self-organized microstructures can feature peaks above or below the initial surface topography [35]. If microstructures grow above the original surface during the structure formation process, primarily due to melt flow and rearrangement of ablated material, they are referred to as above-surface growth mounds (ASG) [34]. If the microstructures remain below the original surface during structure formation, primarily due to PVA, they are referred to as below-surface growth mounds (BSG) [34]. However, apart from the ASG or BSG growth mechanisms, the resulting microstructures are similar in appearance and height-to-width ratio [34]. In addition to fluence, ablation regimes also depend on the material properties, the number of pulses emitted, and the laser wavelength.

While numerous studies have reported the formation of laser-induced surface structures and demonstrated their beneficial effects on specific surface properties, most investigations have focused on limited parameter ranges or individual processing variables. Moreover, a gap remains in the understanding of the fundamental mechanisms that link the processing parameters to the resulting surface features [6]. Consequently, a comprehensive understanding of how the key femtosecond laser processing parameters interact to determine the resulting surface morphology and topography remains incomplete for stainless steel, one of the most frequently used metals in science and industry. In our previous studies on Ti6Al4V and hot-work tool steel, it was shown that an increasing laser pulse overlap lowered the strong ablation threshold more effectively than an increasing scanning line overlap, which was attributed to heat accumulation [33,42,43]. However, it remains unresolved to what extent these process-structure relationships can be transferred to other alloys and which of their aspects are material-specific. This question is particularly relevant for austenitic stainless steel, whose comparatively low thermal conductivity is expected to promote heat accumulation [44]. For this alloy, the influence of the pulse repetition rate has been investigated for a fixed scanning strategy [39], whereas systematic investigations into the effects of laser pulse overlap (PO), scanning line overlap (LO), and fluence on the resulting surface morphology and topography remain lacking. The simultaneous variation in these three parameters is of particular significance, since corresponding PO and LO values result in the same nominal energy input per unit area but different temporal sequences of the laser pulses. This makes it possible to distinguish the effect of the deposited energy from that of its temporal distribution, such as heat accumulation. A detailed understanding of these process–structure relationships is essential for the controlled generation of application-specific surface functionalities. Thus, this study establishes systematic process–structure relationships for femtosecond laser-structured X5CrNi18-10 stainless steel, quantitatively linking PO, LO, and fluence to the resulting surface morphology and topography, and identifying material-specific process windows for controlled surface functionalization.

## 2. Materials and Methods

### 2.1. Material

The experimental investigations in this study were performed on stainless-steel plates (X5CrNi18-10, material designation 1.4301 according to EN 10088 [45], purchased from K&D Handel (Mönchengladbach, Germany). The dimensions of the stainless-steel plates were 78 mm × 78 mm × 1.5 mm. The stainless-steel plates were supplied with a machine-polished surface finish, resulting in an average arithmetic mean height of approximately Sa = 0.027 ± 0.002 µm, determined using the confocal laser scanning microscope described in Section 2.3. Prior to laser texturing, the samples were cleaned with ethanol (purity 99.9%) and dried with dust-free precision wipes and clean, dry compressed air. After the laser treatment, the samples were cleaned in an ultrasonic bath with distilled water for 10 min.

### 2.2. Laser Treatment

**Figure 1:**
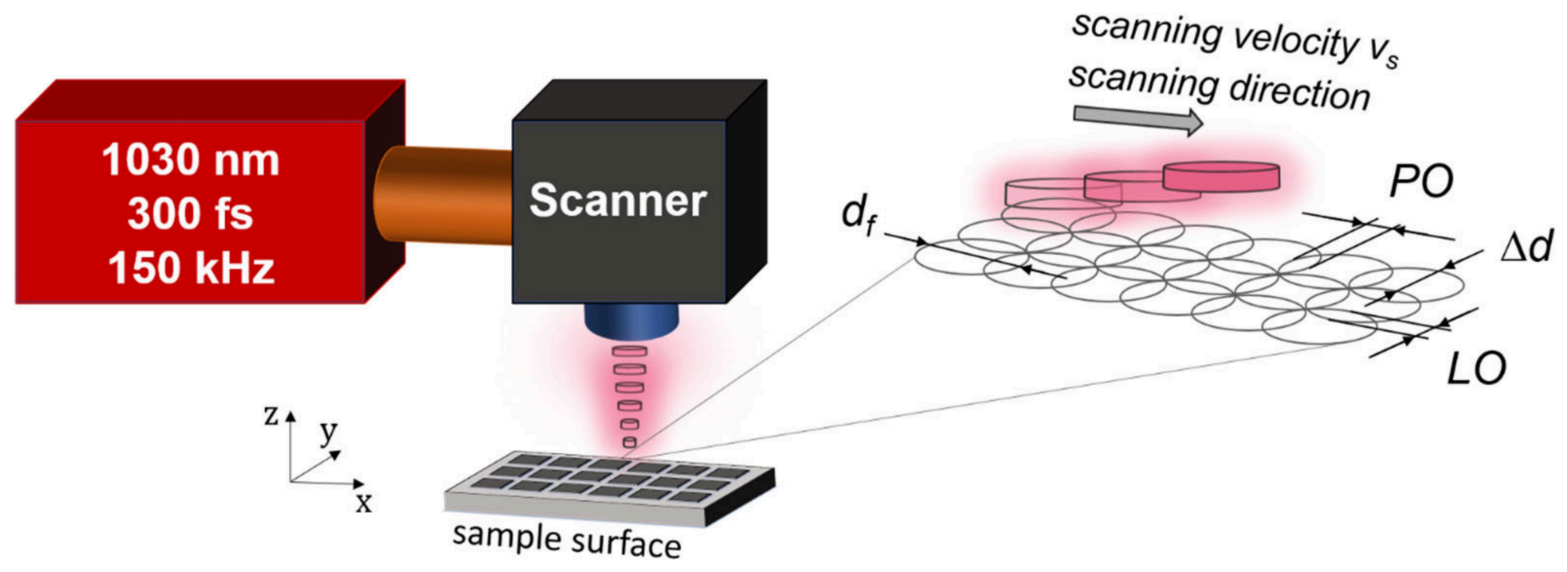
Laser setup and scanning system with the corresponding scanning parameters: theoretical circular focus diameter d_f_, line distance of the scanning lines ∆d, scanning velocity v_s_, laser pulse overlap PO and scanning line overlap LO (Figure by Thomas et al. (2026) [43]. Licensed under CC BY 4.0).

Laser processing was performed using a 300 fs UFFL_60_200_1030_SHG fiber laser (Active Fiber Systems GmbH, Jena, Germany) with a Yb-doped amorphous glass core and a maximum average power of P = 60 W. The pulse repetition rate can be adjusted over the range from f_REP_ = 50.3 kHz to 18.6 MHz. The laser system is integrated into a Microgantry GU4 five-axis micromachining center (Kugler GmbH, Salem, Germany) equipped with an intelliSCAN 14 scanning system (Scanlab GmbH, Puchheim, Germany). Beam deflection in the x- and y-directions was achieved using galvanometer mirrors (Figure 1). The laser emitted linearly polarized radiation at 1030 nm with a Gaussian beam profile. Focusing was performed using an f-theta lens with a focal length of f = 163 mm, resulting in a theoretical circular focal diameter of d_f_ = 31.6 µm at the 1/e^2^ intensity level. This focal diameter was used for all subsequent calculations of the laser processing parameters. During the laser treatment, ablated particles were removed using a high-performance laser fume and particle extraction system positioned near the processing zone. 

As shown in Figure 1, the scanning strategies developed in the planar direction are based on the laser pulse overlap PO and the scanning line overlap LO. The laser pulse overlap is calculated using the following equation:

(1) $$\mathrm{PO}=\left(1-\frac{v_{s}}{d_{f}{\times f}_{\mathrm{REP}}}\right)\times 100\%$$

where v_s_ is the scanning velocity, d_f_ is the theoretical circular focus diameter and f_REP_ the pulse repetition rate. The scanning line overlap LO is calculated using the following equation:

(2) $$\mathrm{LO}=\left(1-\frac{∆d}{d_{f}}\right)\times 100\%$$

where ∆d is the scanning line distance, and d_f_ is the theoretical circular focus diameter, as in Equation (1). The average fluence ϕ is calculated as follows:

(3) $$\phi =\frac{E_{P}}{A}=\frac{E_{P}}{\frac{\pi }{4}\times {d_{f}}^{2}}$$

where E_P_ is the pulse energy, and A is the area of the laser spot diameter with the theoretical circular focus diameter d_f_. Assuming a Gaussian beam profile, the corresponding peak fluence at the beam center is twice the average fluence. The pulse energy E_P_ is calculated using the following equation:

(4) $$E_{P}=P_{m}\times T=\frac{P_{m}}{f_{\mathrm{REP}}}$$

where P_m_ is the average power, T is the time interval between the different pulses, and f_REP_ is the pulse repetition rate. For all experiments, the wavelength λ, pulse duration τ, and pulse repetition rate f_REP_ were kept constant at specific values. In Table 1 these values are shown in addition to the M^2^-factor and the calculated theoretical circular focus diameter d_f_.

To investigate the influence of laser scanning parameters on the morphology and topography of femtosecond laser-structured stainless-steel surfaces, various scanning strategies were defined. Within the framework of these scanning strategies, significant laser scanning parameters were varied and will be discussed in more detail below.

Important parameters for the two-dimensional development of the scanning strategies are, on the one hand, the laser pulse overlap (PO) and, on the other hand, the scanning line overlap (LO), for which various values between 40% and 90% were selected (Table 2). Variation in the laser pulse overlap was achieved by altering the scanning velocity, ranging from *v_s_* = 2.85 m/s to *v_s_* = 0.475 m/s, while scanning line overlap variations were achieved by scanning line feeds ranging from ∆d = 19 µm to ∆d = 3 µm. While one overlap parameter was varied, the second was kept constant at 50%. Alongside laser pulse overlap and scanning line overlap, laser fluence plays a decisive role in ultrashort-pulse laser ablation and material processing. The onset of material removal is governed by the ablation threshold fluence, which depends on the material’s thermal and dynamic response to laser irradiation [46]. Although lower ablation threshold fluences have been reported for stainless steel [47], preliminary experiments conducted in the present study showed that a fluence of approximately ϕ = 0.5 J/cm^2^ corresponds to the onset of homogeneous LIPSS formation under the applied processing conditions. To investigate the full range of surface structures, the average fluence was gradually increased from ϕ = 0.5 J/cm^2^ to ϕ = 17 J/cm^2^ (pulse energy increased from 3.9 µJ to 133.9 µJ) to cover the transition from the initial formation of self-organized nanostructures to the development of pronounced microstructures. In addition to the irradiation wavelength (λ = 1030 nm), the number of overscans (N = 50) was also kept constant throughout this study. Table 2 below lists all laser scanning parameters, including variations.

### 2.3. Surface Characterizations

A confocal laser scanning microscope (CLSM), the LEXT OLS 4000 (Olympus, Hamburg, Germany), was used to measure surface roughness. The average area surface roughness Sa was measured at five different positions (n = 5) on each structured surface, and results are reported as mean ± sample standard deviation. The five measurement positions were predefined according to a fixed scheme comprising the center of each structured field and the centers of its four quadrants, so that the measurements were evenly distributed over the entire processed area. Local defects and irregularities were deliberately not avoided, so that the reported values reflect the actual surface condition, including local inhomogeneities. The roughness measurements were performed using the MPLAPON50XLEXT objective (Olympus, Hamburg, Germany) with a constant optical magnification (50×) and a numerical aperture (NA) of 0.95, resulting in a total magnification of the system of 1080×. The scan area with the objective is 256 μm × 256 μm, and the resulting scans have a resolution of 1024 × 1024 pixels. For data analysis, the OLS4000 software (Version 2.2.3, Olympus, Hamburg, Germany) was used. A cut-off wavelength of λ_C_ = 80 µm was set for all roughness measurements. To obtain high-resolution images and further evaluate the surface morphology after USPL processing, scanning electron microscopy (SEM) was performed using a SUPRA 25 field-emission SEM (Zeiss, Oberkochen, Germany). To quantify the characteristic dimensions of the generated surface structures, the spatial periods Λ_1_ and Λ_2_ were determined from the SEM images using the browser-based image analysis software ImageJ.JS (version 0.6.0, based on ImageJ 1.53m; https://ij.imjoy.io/, accessed in April 2026). Five measurements were performed for each representative structural type. The resulting spatial periods are reported as mean values together with the corresponding standard deviations. In addition, the characteristic lateral dimensions of the micro-crater structures (crater diameter) and of the pillared microstructures (pillar width, measured in top view perpendicular to the longest pillar axis) were determined from the SEM images of the parameter matrices using ImageJ.JS and cross-checked for selected representative fields using CLSM measurements. Owing to the continuous structural evolution within these classes, these dimensions are reported as approximate ranges rather than mean values.

## 3. Results and Discussion

### 3.1. Surface Characterization of Femtosecond Laser-Structured Stainless-Steel Surfaces

**Figure 2:**
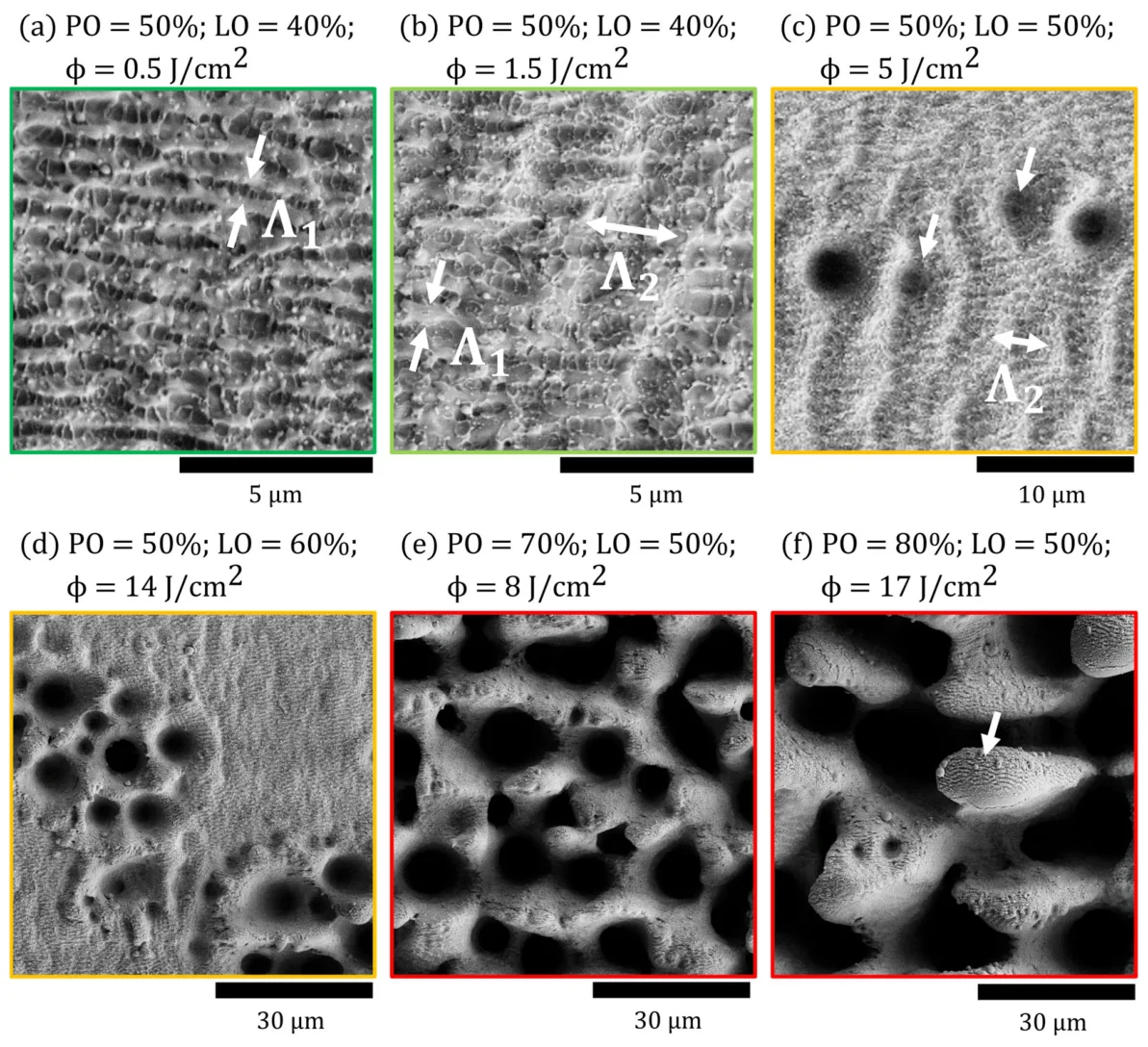
Exemplary SEM images of the resulting surface structures: (**a**) LIPSS with a spatial period Λ_1_(indicated by arrows); (**b**) LIPSS with a spatial period Λ_1_ superimposed with micrometric ripples with a spatial period Λ_2_ (indicated by arrows); (**c**) smaller micro-crater structures (indicated by arrows); (**d**) larger micro-crater structures; (**e**) smaller pillared microstructures; (**f**) larger pillared microstructures, covered with nanoscale LIPSS (indicated by the arrow). Note that different magnifications were selected to represent the typical structural features adequately. The individual surface morphology classes are color-coded for easier tracking in the following Figure 3 and Figure 5.

The structuring experiments described above resulted in the formation of self-organized surface structures exhibiting a wide range of topographical and morphological characteristics. Based on the SEM images, four fundamentally different structure types can be identified. This classification is based on distinct morphological features and follows the evolution sequence reported for femtosecond laser processing of metals with increasing energy input, for example, from LIPSS via micrometric ripples and precursor sites to craters and mound- or pillar-like microstructures [34,35]. The same four structural classes were identified in our previous studies on Ti6Al4V and hot-work tool steel [33,43], and comparable classes have been distinguished using image-based machine-learning classification [48]. The classes are defined by the following criteria: (1) exclusively periodic ripples with a spatial period Λ_1_ close to the irradiation wavelength; (2) LIPSS superimposed by micrometric ripples with a spatial period Λ_2_ of a few micrometers; (3) isolated or clustered crater-like depressions on an otherwise rippled surface; and (4) a continuous three-dimensional relief of pillars separated by deep cavities that covers the entire surface. For quantitative characterization of the morphologically distinguished surface structures, the characteristic dimensions and Sa ranges of the individual structure classes are presented in the following paragraph, together with the representative SEM images in Figure 2a–f. Figure 2 shows SEM images of the different structure types and the progression of structure formation under different average laser fluences and scanning strategies.

**Figure 3:**
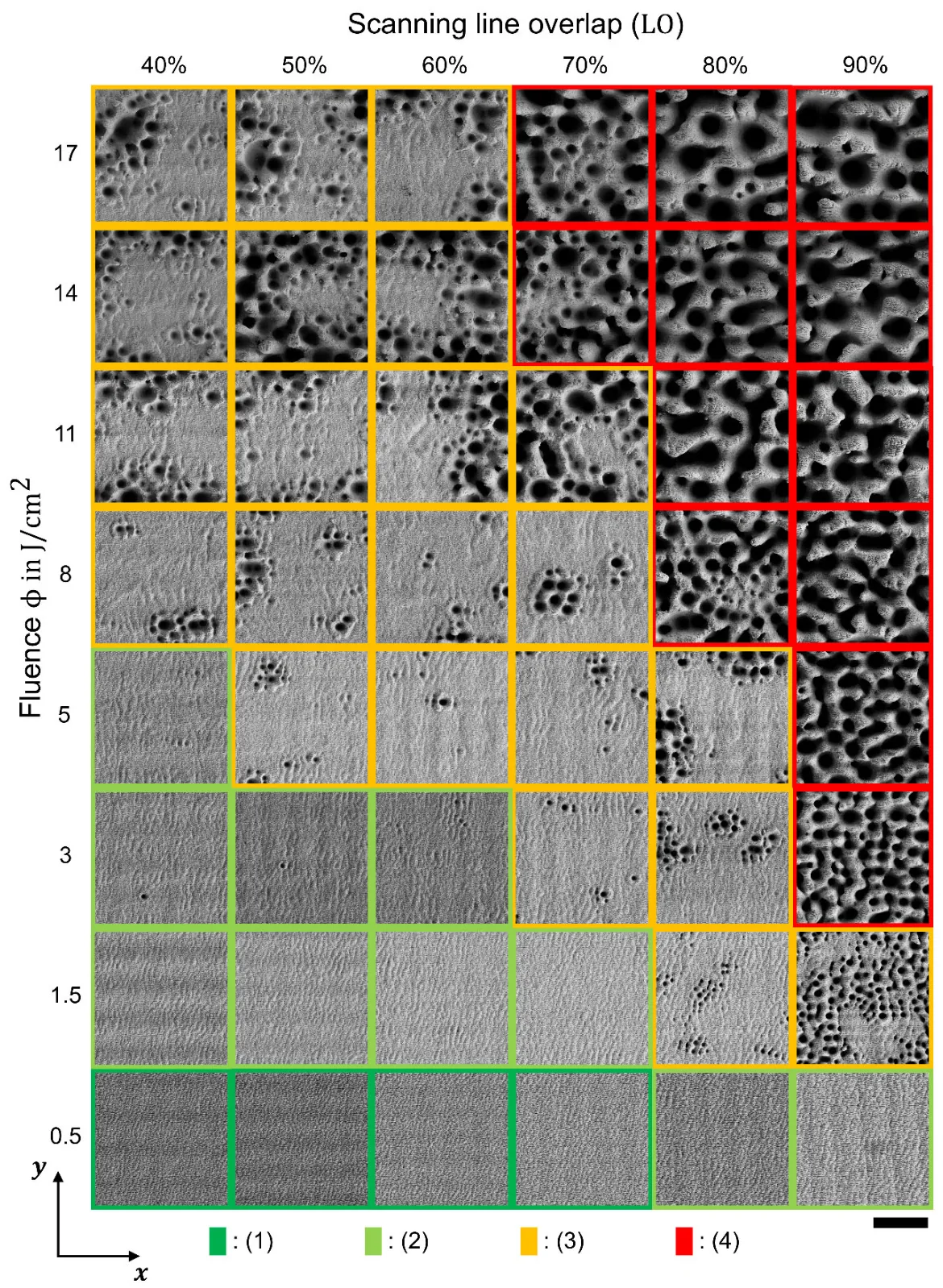
Matrix of SEM images of structured stainless-steel (X5CrNi18-10) surfaces with a fixed laser pulse overlap of PO = 50%, varied scanning line overlap of LO = 40–90%, number of overscans of N = 50, and the average fluence varied between ϕ = 0.5 J/cm^2^–17 J/cm^2^. The scanning lines ran parallel to the x-axis; the offset was changed accordingly in the y-direction. Structure classes: (1)—LIPSS with a spatial period *Λ*_1_; (2)—LIPSS with a spatial period *Λ*_1_ superimposed with micrometric ripples with a spatial period *Λ*_2_; (3)—(smaller and larger) micro-crater structures; (4)—(smaller and larger) pillared microstructures. The scanning direction of the laser beam (and thus the direction for PO) aligns with the x-axis, while the y-axis indicates the direction of the line advance for LO. Scale bar at bottom right corner: 30 µm.

The SEM images in Figure 2 show that the characteristic dimensions of the generated surface structures range from the sub-micrometer scale of the LIPSS (Λ_1_ ≈ 0.78 µm) to pillar widths of up to approximately 30 µm. To simplify the identification of the various structural regimes within the parameter matrix, the SEM images corresponding to the individual structural classes are color-coded consistently in the following figures. The Sa ranges given below for each structure class were compiled from all fields assigned to the respective class in Figure 3 and Figure 5 (Tables S1 and S2). Figure 2a shows the first class of LIPSS, which were generated above the threshold fluence. The LIPSS exemplars shown were generated with a laser pulse overlap of PO = 50%, a laser scanning line overlap of LO = 40%, and an average fluence of ϕ = 0.5 J/cm^2^. Since the LIPSS are aligned perpendicular to the polarization of the laser beam and exhibit a mean spatial period of *Λ*_1_ = 0.784 ± 0.011 µm (labeled in Figure 2a), which is slightly smaller than the irradiation wavelength λ = 1030 nm, the generated structures can be classified as low-spatial-frequency LIPSS (LSFL). All LIPSS observed in this study are LSFL, and the observed alignment and spatial periodicity are consistent with the classical mechanisms of LSFL formation described for metals under femtosecond laser irradiation [5,40]. Surfaces of this first structure class exhibit Sa values between 0.125 µm and 0.266 µm. Figure 2b shows the second structure class of LIPSS with a spatial period Λ_1_ superimposed on micrometric ripples with a mean spatial period of *Λ*_2_ = 3.282 ± 0.099 µm (labeled in Figure 2b). This structural class is hereinafter referred to as a micrometric ripple (MR). The MRs shown were generated with a laser pulse overlap of PO = 50%, a laser scanning line overlap of LO = 40%, and an average fluence of ϕ = 1.5 J/cm^2^. The MRs, generated at an average fluence slightly above that required for gentle ablation, aligned perpendicular to the LIPSS orientation (parallel to the polarization of the laser beam). The observed transition from LSFL-dominated morphologies to superimposed MR structures is consistent with previous reports describing the formation of hierarchical surface morphologies under increasing fluence conditions [33,35,42]. Surfaces of this second structure class exhibit Sa values between 0.158 µm and 0.392 µm, a range that partially overlaps with that of the first class. Figure 2c shows the third structural class of (smaller) micro-crater structures, which were generated with a laser pulse overlap of PO = 50%, a laser scanning line overlap of LO = 50%, and an average fluence of ϕ = 5 J/cm^2^. Compared to the MRs, the average fluence was further increased to trigger crater formation. Figure 2c shows the incipient crater structures forming in the location of the MRs. These smaller micro-crater structures consist of isolated or clustered craters with diameters of approximately 2–5 µm and exhibit Sa values between 0.198 µm and 0.926 µm. Following the emergence of initial inhomogeneities, these features act as localized energy-deposition sites, leading to enhanced absorption and progressive material removal, thereby driving the further evolution of the crater structures [34,35]. Since the average fluence remains comparatively low for the smaller micro-crater structures, and the surface morphology does not exhibit clear signatures of pronounced thermal effects, the PVA growth mechanism appears to dominate. This behavior is consistent with the structure-evolution mechanisms described in previous studies [33,35,42], in which local topographical variations promote energy deposition and enhanced ablation. Consequently, it can be assumed that reflections from the side walls of the MRs increase the local laser light fluence, thereby promoting continuous local growth of micro-crater structures. This phenomenon is obvious in the surrounding inhomogeneities in Figure 2c, as these crater structures have already grown larger than the marked inhomogeneities. Figure 2d shows a larger micro-crater structure in which the crater structures have already grown significantly. These larger micro-crater structures were generated with a laser pulse overlap of PO = 50%, a laser scanning line overlap of LO = 60%, and an average fluence of ϕ = 14 J/cm^2^. The larger micro-crater structures are characterized by enlarged and partly coalesced craters with diameters of approximately 5–15 µm and exhibit Sa values between approximately 1.1 µm and 3.188 µm. Since a higher average fluence was applied than for the smaller micro-crater structures, the observed morphological evolution may suggest a transition toward formation mechanisms involving increased thermal contributions. Similar morphological features and comparable structures have been attributed in the literature to localized heat accumulation, melt dynamics, and vapor–liquid–solid growth processes [35,42]. The pronounced morphological evolution observed at higher fluences is consistent with morphological trends reported in previous studies, in which increasing fluence and cumulative energy deposition were found to promote thermally induced restructuring processes [35,42]. Figure 2e shows the fourth structural class of (smaller) pillared microstructures, which were generated with a laser pulse overlap of PO = 70%, a laser scanning line overlap of LO = 50%, and an average fluence of ϕ = 8 J/cm^2^. The smaller pillared microstructures, which form at the onset of this class, exhibit pillar widths of approximately 3–15 µm, increasing with fluence, and Sa values between 1.299 µm and approximately 5.5 µm. The planar morphology of the pillared microstructures was created by exceeding the strong ablation threshold with the scanning strategy used. If the fluence is further increased, the smaller pillared microstructures become more pronounced. Figure 2f shows larger pillared microstructures generated with a laser pulse overlap of PO = 80%, a laser scanning line overlap of LO = 50%, and an average fluence of ϕ = 17 J/cm^2^. The larger pillared microstructures exhibit pillar widths of approximately 10–30 µm and reach Sa values of up to 6.896 µm when LO is varied and up to 13.463 µm when PO is varied. These pillared microstructures are partly covered by nanostructures (see the arrow in Figure 2f), particularly LIPSS, on the structural peaks, as a result of the Gaussian intensity profile of the laser beam and the consequently significantly lower fluence at the outer edge of the profile. The observation of microstructures covered with nanoscale LIPSS is consistent with the formation of hierarchical surface morphologies described in the literature [33]. The LIPSS on the structural peaks of the pillared microstructures are labeled in Figure 2f. Since the Sa ranges of adjacent structure classes partially overlap, the classification is based on the morphological criteria described above, and Sa serves as a complementary quantitative descriptor of the structure classes.

### 3.2. Effect of Scanning Line Overlap on Topography and Morphology

To investigate the effect of the scanning line overlap at different laser fluence levels on the topography and morphology of stainless-steel surfaces, the overlap was varied between LO = 40% and LO = 90% with a fixed laser pulse overlap of PO = 50%. The results of the parameter study of the structured stainless-steel (X5CrNi18-10) surfaces with a fixed laser pulse overlap of PO = 50% and a varied scanning line overlap are shown in Figure 3.

The rows of the SEM matrix in Figure 3 correspond to the applied average fluences (ϕ = 0.5–17 J/cm^2^) and the columns to the specified scanning line overlaps (LO = 40–90%). In this study on the effect of scanning line overlap on topography and morphology, the parameters for the laser pulse overlap and the number of overscans were kept constant (PO = 50%; N = 50).

Based on the resulting morphology and topography of the generated laser-induced surface structures, these can be classified into the four basic structural classes introduced and described in the previous Section 3.1. In the SEM matrix shown, the generated structural classes are color-coded for clarity. The first class of LIPSS with a spatial period Λ_1_ is formed in the laser scanning line overlap range from LO = 40% to LO = 70% at an average fluence of ϕ = 0.5 J/cm^2^. All LIPSS generated in this matrix to investigate the effect of scanning line overlap are oriented perpendicular to the polarization of the laser beam, and their spatial period Λ_1_ is equal to or slightly smaller than the irradiation wavelength. Thus, the structures generated here are LIPSS with low spatial frequency (LSFL). The observed orientation and spatial periodicity are in accordance with the known characteristics of LSFL formation on metallic surfaces under femtosecond laser irradiation, which results from interference effects between the incident laser beam and the surface electromagnetic waves, among other material–light interactions depending on the laser fluence [5,40]. As a second structure class, LIPSS with a spatial period Λ_1_ superimposed with micrometric ripples with a spatial period Λ_2_ were identified. Compared to the first structure class of LIPSS, LIPSS superimposed with MR form at a higher scanning line overlap or a higher average fluence. The observed transitions from LSFL-dominated morphologies to hierarchical MR structures under conditions of increased fluence have previously been described in the context of femtosecond laser processing of metallic materials [4,5]. For the range of higher scanning line overlap, LIPSS superimposed with MR are formed in the laser scanning line overlap range of LO = 80% to LO = 90% at an average fluence of ϕ = 0.5 J/cm^2^. For the higher fluence range, LIPSS superimposed with MR are formed in the laser scanning line overlap range of LO = 40% to LO = 70% at an average fluence of ϕ = 1.5 J/cm^2^, in the laser scanning line overlap range of LO = 40% to LO = 60% at an average fluence of ϕ = 3 J/cm^2^ and at a laser scanning line overlap of LO = 40% at an average fluence of ϕ = 5 J/cm^2^. The MR features can therefore be regarded as precursor cells from which smaller and larger micro-crater structures develop at higher scanning line overlap or higher average fluence. This morphological transition suggests a progressive change in the material–light interaction with increasing local energy input. Even at an average fluence of ϕ = 1.5 J/cm^2^, the first smaller micro-crater structures form at a laser scanning line overlap of LO = 80% and LO = 90%. If the average fluence is increased to ϕ = 3 J/cm^2^, smaller micro-crater structures form at a laser scanning line overlap of LO = 70% and LO = 80%. Starting at an average fluence of ϕ = 5 J/cm^2^, the smaller micro-crater structures form within a wide laser scanning line overlap range of LO = 50% to LO = 80%. At higher average fluences starting at ϕ = 8 J/cm^2^, the smaller micro-crater structures continue to coalesce into larger micro-crater structures as the average fluence increases. The number and size of the craters increase with increasing fluence and increasing laser scanning line overlap. The observed increase in crater number and crater size with increasing fluence and scanning line overlap is consistent with previous studies on femtosecond laser processing of metals, which reported a progressive evolution from periodic surface structures to localized crater formation as the deposited energy increases [34,35]. Furthermore, the formation process of craters has already been described for femtosecond laser processing of metals, in which local topographical inhomogeneities promote preferential absorption and enhanced ablation at specific locations on the surface [34]. In the high-fluence ranges, the micro-crater structures become increasingly pronounced, accompanied by a substantial increase in crater size and density. This morphological evolution suggests a shift in the dominant mechanism of structure formation, compared to the smaller micro-crater structures observed at lower fluences. Similar morphological developments have been associated with increased cumulative energy deposition and thermally influenced restructuring processes during femtosecond laser processing of metallic materials [42]. The larger micro-crater structures form even in the highest average fluence range of ϕ = 17 J/cm^2^ at a lower laser scanning line overlap range of LO = 40% to LO = 60%. The fourth structural class of smaller and larger pillared microstructures also arises from the crater structures at higher scanning line overlap or higher fluence. The observed evolution toward pillared microstructures resembles the below-surface growth (BSG) morphologies reported by Zuhlke et al. [34], in which preferential material removal in the surrounding regions leads to the apparent inward growth of the pillar structures. In the low average fluence range of ϕ = 3 J/cm^2^ and ϕ = 5 J/cm^2^, the first smaller pillared microstructures form at the highest laser scanning line overlap of LO = 90%. If the average fluence is then increased to ϕ = 8 J/cm^2^, the smaller pillared microstructures form even at a laser scanning line overlap of LO = 80%. In the high average fluence range of ϕ = 14 J/cm^2^ and ϕ = 17 J/cm^2^, starting at a scanning line overlap of LO = 70%, the strong ablation threshold is already exceeded, and consequently, less pronounced pillared microstructures form from this point onward. Similar morphology transitions above the strong ablation threshold have been reported for ultrashort-pulse laser processing of metals and are generally associated with enhanced material removal rates and pronounced thermally assisted restructuring effects [4]. With increasing fluence and scanning line overlap, the structures progressively evolve into larger pillared microstructures, accompanied by an increase in the characteristic dimensions of the individual pillars. This observation further demonstrates the strong dependence of hierarchical surface structure evolution on the combined influence of local energy deposition and cumulative thermal effects.

**Figure 4:**
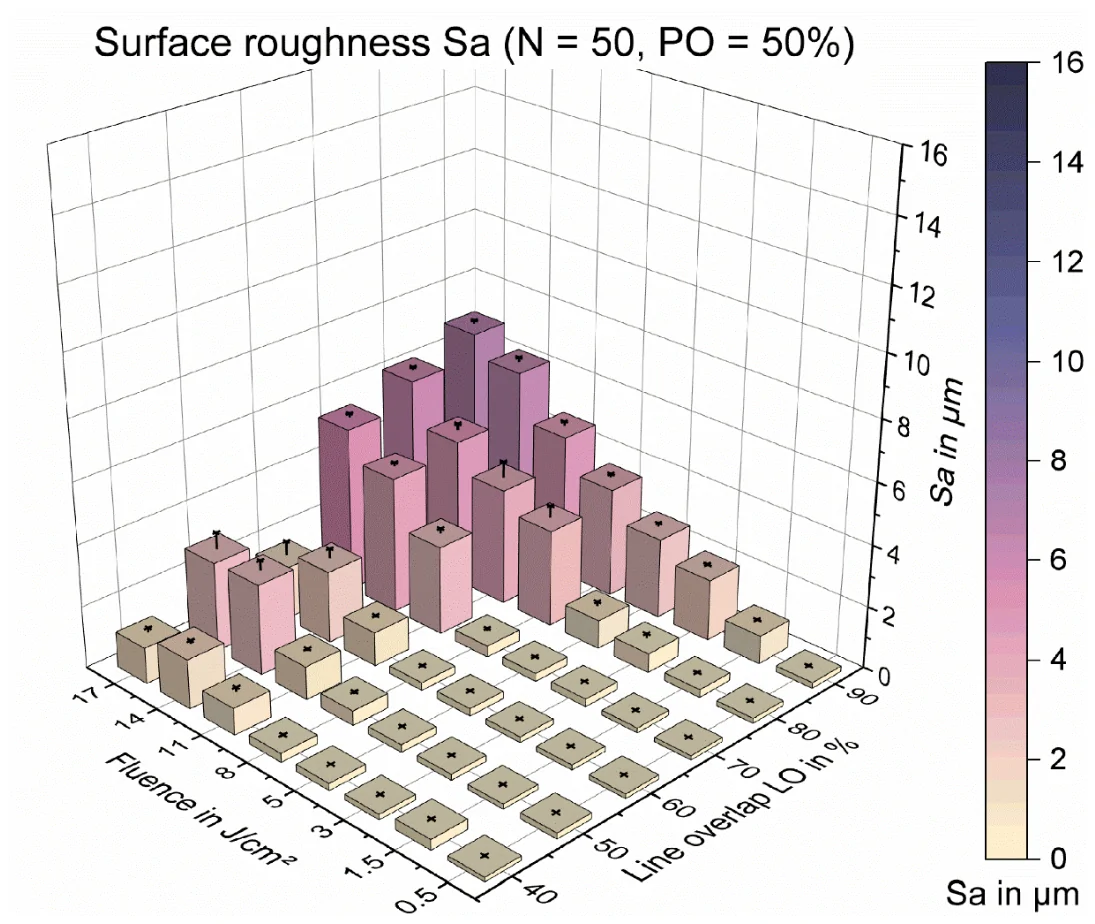
Matrix of surface roughness values Sa (arithmetical mean height) of structured stainless-steel (X5CrNi18-10) surfaces with a fixed laser pulse overlap of PO = 50%, varied scanning line overlap of LO = 40–90%, number of overscans of N = 50, and the average fluence varied between ϕ = 0.5 J/cm^2^–17 J/cm^2^.

For further surface characterization, Figure 4 below shows the average surface roughness data Sa associated with the parameter matrix (see Figure 3). The correlation between structural evolution and roughness development provides additional insight into the effect of fluence and scanning line overlaps on the resulting functional surface characteristics. The surface roughness data is presented as a three-dimensional bar chart that maps each roughness value to the corresponding field in the parameter matrix shown in Figure 3.

At the lowest investigated average fluence of ϕ = 0.5 J/cm^2^, the measured surface roughness values remain relatively low, with a minimum value of Sa = 0.141 µm ± 0.001 µm observed at a scanning line overlap of LO = 40%. For the first structure class of LIPSS with a spatial period Λ_1_, the surface roughness values extend up to Sa = 0.191 µm ± 0.001 µm. For the second structure class of LIPSS with a spatial period Λ_1_ superimposed with micrometric ripples with a spatial period Λ_2_, the surface roughness values range from Sa = 0.159 µm ± 0.003 µm to Sa = 0.331 µm ± 0.005 µm. The moderate increase in surface roughness associated with MR formation indicates the onset of hierarchical surface structuring driven by increased local energy deposition. This trend is consistent with previous observations of multiscale femtosecond laser-induced surface structures reported in the literature [5]. For both structure classes, the roughness values remain comparatively homogeneous across the investigated parameter range, suggesting stable LSFL-dominated and MR-dominated formation conditions within the gentle ablation regime. The comparatively low roughness values observed in this parameter range are attributed to material removal in the gentle ablation regime, where limited thermal influence and reduced melt formation result in comparatively smooth surface morphologies. Similar behavior has previously been reported for femtosecond laser processing of metallic materials at fluences slightly above the ablation threshold [4,5]. In the range of the third structure class, the roughness values then gradually increase up to the larger micro-crater structures, reaching a surface roughness value of Sa = 2.929 µm ± 0.304 µm at an average fluence of ϕ = 14 J/cm^2^ and a scanning line overlap of LO = 50%. This progressive increase in roughness correlates with the transition from LIPSS-dominated morphologies to increasingly pronounced crater formation, reflecting enhanced localized energy deposition and material redistribution. A similar roughness evolution associated with crater coalescence and thermally enhanced structure growth has previously been reported under elevated cumulative energy deposition conditions during femtosecond laser processing of Ti6Al4V [42]. The abrupt increase in roughness observed for the fourth structure class indicates that the strong ablation threshold has been exceeded, resulting in intensified material removal and the formation of pronounced three-dimensional surface morphologies. In this structure class, the surface roughness values range from Sa = 1.987 µm ± 0.043 µm for the smaller microstructures to Sa = 6.896 µm ± 0.092 µm for the maximum value in the larger microstructure at an average fluence of ϕ = 17 J/cm^2^ and a scanning line overlap of LO = 90%. The abrupt increase in roughness observed for the fourth structure class coincides with the transition from micro-crater structures to pronounced pillared microstructures. This finding indicates the development of increasingly pronounced three-dimensional surface features. Similar morphology-dependent roughness increases have been reported in previous studies on femtosecond laser structuring of metals, where the evolution from periodic nanostructures to micro-structured surface topographies resulted in markedly higher roughness values [4,5,33,43]. The complete surface roughness values for Sa for the entire matrix can be found in Table S1 in the Supplementary Materials.

### 3.3. Effect of Laser Pulse Overlap on Topography and Morphology

**Figure 5:**
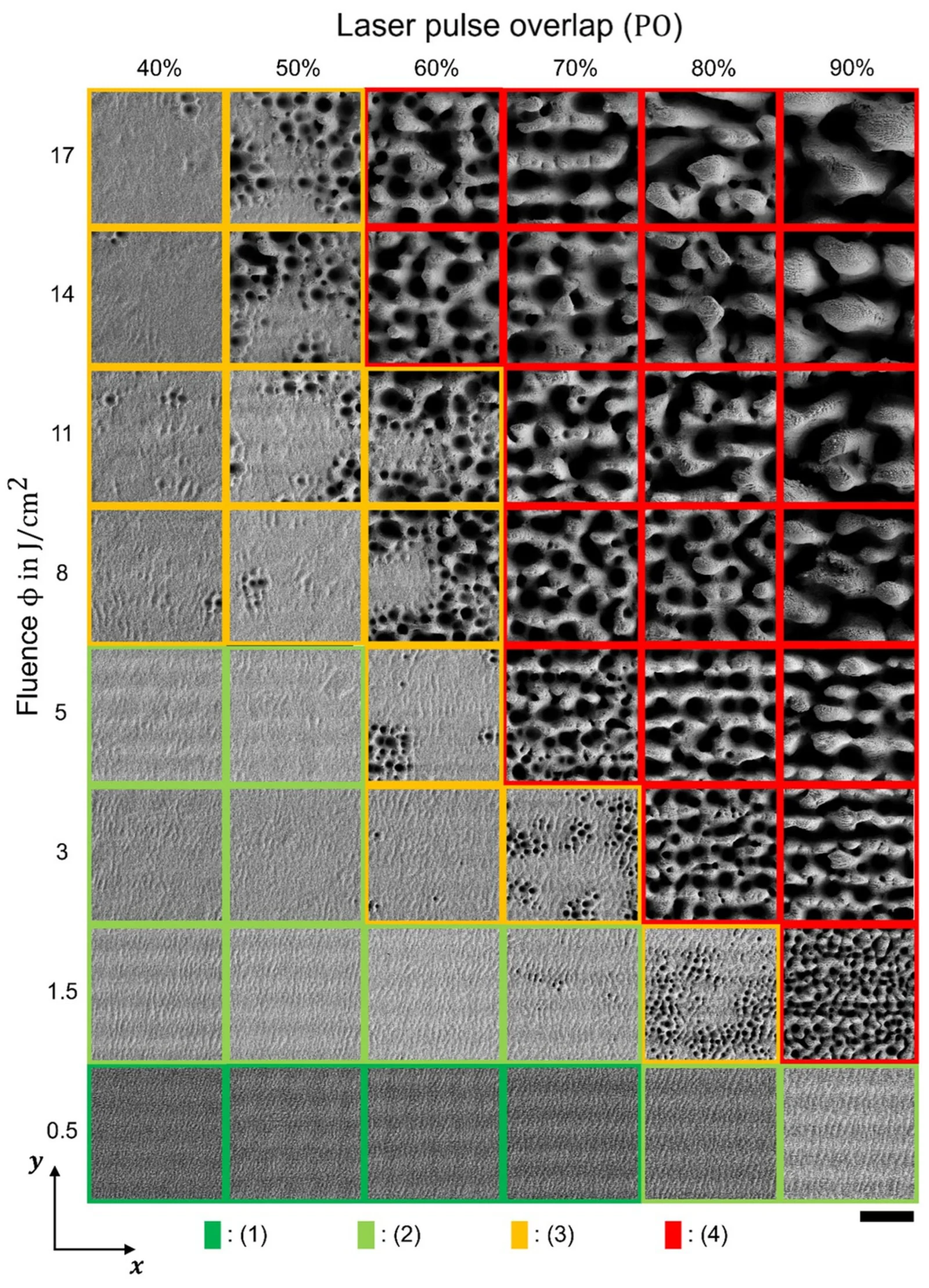
Matrix of SEM images of structured stainless-steel (X5CrNi18-10) surfaces with a varied laser pulse overlap of PO = 40–90%, fixed scanning line overlap of LO = 50%, number of overscans of N = 50, and the fluence varied between ϕ = 0.5 J/cm^2^–17 J/cm^2^. The x-axis indicates the scanning direction for the laser pulse overlap PO. Structure classes: (1)—LIPSS with a spatial period *Λ*_1_; (2)—LIPSS with a spatial period *Λ*_1_ superimposed with micrometric ripples with a spatial period *Λ*_2_; (3)—(smaller and larger) micro-crater structures; (4)—(smaller and larger) pillared microstructures. The scanning direction of the laser beam (and thus the direction for PO) aligns with the x-axis, while the y-axis indicates the direction of the line advance for LO. Scale bar at bottom right corner: 30 µm.

For the second sample, laser pulse overlap was varied between PO = 40% and PO = 90% with a constant scanning line overlap of LO = 50% to investigate the effect of laser pulse overlap on the topography and morphology of femtosecond laser-structured stainless-steel surfaces. The results of the parameter study with a fixed scanning line overlap and a varied laser pulse overlap are shown in Figure 5.

As before, the rows of the SEM matrix in Figure 5 correspond to the investigated average fluence range (ϕ = 0.5–17 J/cm^2^), whereas the columns represent the applied laser pulse overlaps (PO = 40–90%). In this matrix on the effect of laser pulse overlap on topography and morphology, the parameters for the scanning line overlap and the number of overscans were kept constant (LO = 50%; N = 50). Based on the resulting morphology and topography, the generated laser-induced surface structures were also classified into the four basic structure classes and color-coded.

The first class of LIPSS with a spatial period Λ_1_ is formed at an average fluence of ϕ = 0.5 J/cm^2^, just above the ablation threshold, in the laser pulse overlap range of PO = 40% to PO = 70%. The second structure class of LIPSS with a spatial period Λ_1_ superimposed with micrometric ripples with a spatial period Λ_2_ formed at a higher laser pulse overlap or a higher fluence compared to the first structure class. Compared with the scanning line overlap study, MR structures appear at lower fluences when the laser pulse overlap is increased. This observation indicates that increasing PO is more effective in ablation due to temporally concentrated energy deposition along the scanning direction, thereby accelerating the transition from LSFL-dominated morphologies toward hierarchical surface structures. For the range of higher laser pulse overlap, LIPSS superimposed with MR form at a low average fluence of ϕ = 0.5 J/cm^2^ at a laser pulse overlap of PO = 80% to PO = 90%. At higher fluences, LIPSS superimposed with MR form in the laser pulse overlap range of PO = 40% to PO = 70% at an average fluence of ϕ = 1.5 J/cm^2^ and for average fluences of ϕ = 3 J/cm^2^ and ϕ = 5 J/cm^2^ for the laser pulse overlap range of PO = 40–50%.

At higher fluences and laser pulse overlaps, the MR structures progressively evolve into smaller and subsequently larger micro-crater structures, which constitute the third structure class. Within this structure class, when varying the fluence and laser pulse overlap in this matrix, mainly smaller micro-crater structures form in the narrow corridor, starting at a laser pulse overlap of PO = 80% at an average fluence of ϕ = 1.5 J/cm^2^, over a range from a laser pulse overlap of PO = 60–70% at an average fluence of ϕ = 3 J/cm^2^ up to a maximum average fluence of ϕ = 17 J/cm^2^ and a low laser pulse overlap of PO = 40%. The number and size of the craters increase with increasing fluence and increasing laser pulse overlap. Larger micro-crater structures form in this matrix only in the range of average fluences ϕ = 8–11 J/cm^2^ and a laser pulse overlap of PO = 60% as well as at an average fluence of ϕ = 14–17 J/cm^2^ and a laser pulse overlap of PO = 50%. In these high-fluence regimes, a transition in the dominant crater growth mechanism can be observed, shifting from PVA-dominated growth toward thermally driven crater evolution caused by cumulative energy accumulation at high laser pulse overlap. Similar transitions toward thermally dominated morphology evolution under conditions of elevated pulse accumulation and heat accumulation have previously been reported for high-repetition-rate femtosecond laser processing of metallic materials [42]. In contrast to scanning line overlap, which primarily influences the homogeneity and spatial distribution of the generated structures, an increased laser pulse overlap promotes local heat accumulation along the scanning direction, particularly at higher repetition rates leading to vapor–liquid–solid growth and pronounced melt flow in the inhomogeneous regions. This can be attributed to the different temporal sequence of the laser pulses. Along the scanning line, a given location is irradiated by successive pulses that are separated only by the time interval *T* between two pulses (Equation (4)), and the number of these pulses increases with PO. As previously reported for femtosecond laser processing of Ti6Al4V at high pulse repetition rates [42], relatively short time intervals *T* do not allow the residual heat remaining in the material after each pulse to dissipate, resulting in heat accumulation in the irradiated area. In contrast, the additional pulses resulting from an increased LO are delivered by adjacent scanning lines, which irradiate the same location only after the preceding scanning line has been completed, so that the residual heat can largely dissipate in between. Accordingly, an equivalent nominal accumulated energy does not necessarily result in an equivalent surface response when the number of overscans, pulse fluence, or laser pulse overlap is varied, because pulse sequence, incubation effects, and heat-accumulation effects modify the evolution of the surface structures [5,42].

The fourth structure class, smaller and larger pillared microstructures, arises from crater structures at higher laser pulse overlap or higher fluence. The first smaller pillared microstructures already form in the low fluence range of ϕ = 1.5 J/cm^2^ at a high laser pulse overlap of PO = 90%, in contrast to the comparatively same level of energy input per area as used before (LO = 90%). This observation further highlights the pronounced influence of pulse overlap on cumulative thermal effects and strong-ablation-induced structure evolution. Increasing fluence and laser pulse overlap promote progressive pillar growth and morphological coarsening, indicating intensified cumulative energy accumulation and thermally assisted restructuring processes. For example, at an average fluence of ϕ = 5 J/cm^2^ they form already at a laser pulse overlap of PO = 70%. At the highest average fluence of ϕ = 17 J/cm^2^ the smaller pillared microstructures form already at a laser pulse overlap of PO = 60%. With increasing laser fluence and pulse overlap, the surface morphology progressively evolves toward larger pillared microstructures, accompanied by an increase in the characteristic dimensions of the individual pillars.

**Figure 6:**
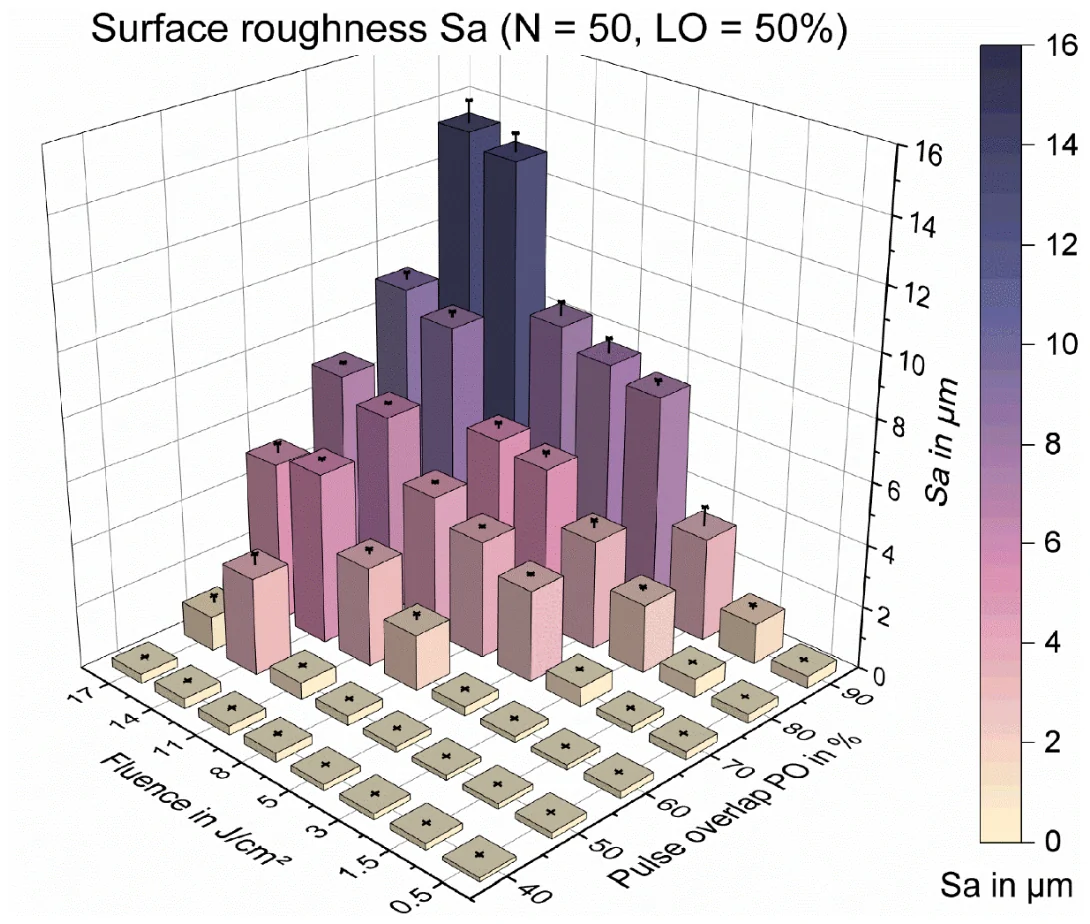
Matrix of surface roughness values Sa (arithmetical mean height) of structured stainless-steel (X5CrNi18-10) surfaces with a fixed scanning line overlap of LO = 50%, varied laser pulse overlap of PO = 40–90%, number of overscans of N = 50, and the average fluence varied between ϕ = 0.5 J/cm^2^–17 J/cm^2^.

The observed morphology transitions are further reflected in the corresponding surface roughness evolution discussed below. For further surface characterization, Figure 6 below shows the surface roughness data Sa associated with the parameter matrix (see Figure 5). The surface roughness data is presented as a three-dimensional bar chart, which maps the respective roughness value to the corresponding matrix fields in the parameter matrix from Figure 5.

The bar chart shows very low surface roughness values for the first structure class of LIPSS with a spatial period Λ_1_, ranging from Sa = 0.125 µm ± 0.058 µm to Sa = 0.266 µm ± 0.002 µm. For the second structure class of LIPSS with a spatial period Λ_1_ superimposed with micrometric ripples with a spatial period Λ_2_, the surface roughness values range from Sa = 0.158 µm ± 0.004 µm to Sa = 0.392 µm ± 0.004 µm. The comparatively low roughness values observed for the first and second structure classes are attributed to material removal within the gentle ablation regime, where limited thermal influence and no or reduced melt formation result in comparatively smooth surface morphologies. This observation is consistent with previous studies describing comparatively smooth LSFL and MR-dominated morphologies generated slightly above the ablation threshold [4,5]. Compared with the matrix obtained by varying the scanning line overlap, the transition from LSFL- and MR-dominated morphologies to micro-crater structures occurs at lower fluences as the laser pulse overlap increases. This observation indicates that laser pulse overlap exerts a stronger influence on structure evolution and surface roughness than scanning line overlap under the investigated processing conditions. For the third structure class of smaller and larger micro-crater structures, the roughness values then increase gradually from a surface roughness value of Sa = 0.198 µm ± 0.007 µm at an average fluence of ϕ = 3 J/cm^2^ and a laser pulse overlap of PO = 60%. This progressive increase in roughness correlates with the transition from ripple-dominated morphologies toward crater-mediated surface evolution and reflects increasingly localized energy deposition and material redistribution. Similar roughness increases associated with crater coalescence and thermally enhanced structure growth under elevated pulse accumulation conditions have previously been reported during femtosecond laser processing [42]. For the larger micro-crater structures, the highest surface roughness value is Sa = 3.188 µm ± 0.163 µm at an average fluence of ϕ = 11 J/cm^2^ and a laser pulse overlap of PO = 60%. The abrupt increase in roughness observed for the fourth structure class of smaller and larger microstructures indicates that the strong ablation threshold has already been exceeded, resulting in intensified material removal and the formation of pronounced three-dimensional surface morphologies. The surface roughness values here range from Sa = 1.299 µm ± 0.139 µm for the smaller microstructures to a maximum value of Sa = 13.463 µm ± 0.702 µm for the larger microstructure at an average fluence of ϕ = 17 J/cm^2^ and a laser pulse overlap of PO = 90%. The observed abrupt increase in roughness above the strong ablation threshold is consistent with previous reports describing thermally assisted restructuring, enhanced melt flow, and vapor–liquid–solid growth under conditions of increased cumulative energy deposition [4,42]. A comparison with results obtained by varying the scanning line overlap reveals that increasing the laser pulse overlap leads to substantially higher roughness values and promotes the formation of crater and pillared microstructures at lower fluences. Whereas the maximum roughness obtained in the scanning-line-overlap matrix was Sa = 6.896 µm ± 0.092 µm, the laser-pulse-overlap matrix yielded a maximum roughness of Sa = 13.463 µm ± 0.702 µm. These findings demonstrate that laser pulse overlap is the more influential parameter governing the evolution of surface topography under the investigated processing conditions. Overall, the roughness analysis confirms that laser pulse overlap has a stronger effect on both morphology evolution and resulting surface roughness than scanning line overlap, leading to an earlier onset of complex microstructures and substantially higher roughness values across the investigated fluence range. The complete surface roughness values for Sa for the entire matrix can be found in Table S2 in the Supplementary Materials.

## 4. Conclusions

In this study, the effects of key laser scanning parameters and fluence on the formation of laser-induced, self-organized surface structures, as well as the resulting topography and morphology on stainless-steel surfaces, were comprehensively investigated. Extensive surface roughness measurements were performed to examine the topography. The morphology was analyzed using a wide range of SEM images of the parameter matrices. Within the scope of the topographical and morphological investigations, four distinct structural types were identified based on the parameters examined.

In the lower region of the matrices, the first structure class generated was LIPSS with a spatial period Λ_1_. The second structure class identified was LIPSS with a spatial period Λ_1_ superimposed with micrometric ripples having a spatial period Λ_2_. Furthermore, smaller and larger micro-crater structures as well as pillared microstructures could be generated. Typically, the pillared microstructures are superimposed with nanostructures, resulting in a double-scale formation. When summarizing all results, the following main findings can be made:

- All investigated laser parameters and scanning strategies have a significant influence on the generation of the self-organized, laser-induced surface structures.
- It was shown that the strong ablation threshold depends on the laser pulse overlap, the scanning line overlap, as well as on the fluence.
- When laser pulse overlaps are varied, smaller and larger microstructures form at lower fluences than when the scanning line overlaps are varied, and these microstructures also exhibit significantly higher roughness. Since corresponding PO and LO values result in the same nominal energy input per unit area, this is attributed to stronger heat accumulation owing to the short time interval between successive pulses along the scanning line.
- Varying the laser pulse overlap and fluence results in significant variation in the structural characteristics of the smaller and larger microstructures and their roughness values.
- Since corresponding PO and LO values result in the same nominal energy input and number of pulses per unit area, and thus in the same theoretical processing time, a relatively high PO should be preferred to efficiently generate pronounced microstructures and high surface roughness according to the roughness analysis performed in this study. The earlier transition to the strong ablation regime, which is characterized by considerably higher ablation rates [4,37], and the formation of pillared microstructures, which requires the removal of material between the pillars, indicate increased material removal at higher PO, in agreement with the conclusions of previous studies [33,43]. However, since the material removal rate was not quantified directly in this study, this should be verified in future work, for example by ablation depth measurements.
- To efficiently achieve high roughness with the lowest possible fluence, high overlaps should be used (at a fixed LO = 50%, POs from 70 to 90%; and at a fixed PO = 50%, LOs from 70 to 90%).

Our comprehensive parametric study on the influence of scanning line overlap, laser pulse overlap, and fluence on topography and morphology provides a fundamental understanding of the structural design of laser-induced, self-organized structures on stainless-steel surfaces. The findings of this study can therefore be used to develop customized structures for various applications. For instance, the LIPSS- and ripple-dominated morphologies obtained here at low fluence and pulse overlap are topographically comparable to those reported for friction-reducing, tribologically optimized stainless-steel surfaces [10,22]. Similarly, the hierarchical structures identified in this study are consistent with those used to tailor surface wettability on stainless steel, ranging from hydrophilic to superhydrophobic behavior [24,25,26,27]. Thus, by systematically linking PO, LO, and fluence to these distinct, well-defined structure classes, the present dataset provides a practical basis for selecting appropriate processing conditions to target such tribological and wetting-related functionalities. Therefore, the controlled generation of application-specific surface structures demonstrated in this study directly supports these established applications of stainless steel and provides a basis for controlling laser processing parameters and further developing quality assurance systems [48].

## Figures and Tables

**Table 1:** Laser source and beam parameters.

Parameter	Symbol	Unit	Value
Wavelength	λ	nm	1030 (IR)
Theoretical circular focus diameter (1/*e*^2^ intensity level)	d_f_	µm	31.6
Pulse duration	τ	fs	300
Pulse repetition rate	f_REP_	kHz	150
*M*^2^-factor (beam quality)	*M* ^2^	-	1.2

**Table 2:** Laser scanning parameters and variations. Laser pulse overlap (PO) and scanning line overlap (LO) were varied for each pulse energy/fluence value.

Laser Scanning Parameter + Variations	Symbol	Unit	Values
Laser pulse overlap (at fixed *LO* of 50%)	*PO*	-	40%; 50%; 60%; 70%; 80%; 90%
Scanning velocity	*v_s_*	m/s	2.850; 2.375; 1.900; 1.425; 0.950; 0.475
Scanning line overlap (at fixed *PO* of 50%)	*LO*	-	40%; 50%; 60%; 70%; 80%; 90%
Scanning line distance	∆*d*	µm	19; 16; 13; 10; 6; 3
Pulse energy	E_P_	µJ	3.9; 11.8; 23.6; 39.3; 63.0; 86.6; 110.2; 133.9
Average fluence	ϕ	J/cm^2^	0.5; 1.5; 3.0; 5.0; 8.0; 11.0; 14.0; 17.0
Peak fluence	ϕ*_peak_*	J/cm^2^	1.0; 3.0; 6.0; 10.0; 16.0; 22.0; 28.0; 34.0
Number of overscans	N	-	50

## Data Availability

The original contributions presented in this study are included in the article/Supplementary Materials. Further inquiries can be directed to the corresponding author.

## Supplementary Materials

The following supporting information can be downloaded at: https://www.mdpi.com/article/10.3390/mi17101189/s1, Table S1: Surface area roughness Sa (N = 50, PO = 50%); Table S2: Surface area roughness Sa (N = 50, LO = 50%).

## Abbreviations

The following abbreviations are used in this manuscript:

ASG	Above-Surface Growth
BSG	Below-Surface Growth
CLSM	Confocal Laser Scanning Microscope
HSFL	High-Spatial-Frequency Laser-Induced Periodic Surface Structures
LIPSS	Laser-Induced Periodic Surface Structures
LO	Scanning Line Overlap
LSFL	Low-Spatial-Frequency Laser-Induced Periodic Surface Structures
MR	(Sub-)Micrometric Ripple(s)
NA	Numerical Aperture
PVA	Preferential Valley Ablation
PO	Laser Pulse Overlap
SEM	Scanning Electron Microscope/Scanning Electron Microscopy
USPL	Ultrashort-Pulse Laser
VLS	Vapor–Liquid–Solid
