# Supplementary material for: Effect of Laser Fluence and Scanning Strategy on Topography and Morphology of Femtosecond Laser-Structured Stainless-Steel Surfaces
Source: Micromachines (Basel). 2026 Oct 14;17(10):1189. doi: 10.3390/mi17101189 (PMC13707821; doi:10.3390/mi17101189)
Supplement: Supplementary file 1 [file micromachines-17-01189-s001.zip › micromachines-4566180-supplementary.pdf]

Table S1. Surface area roughness Sa (N = 50, PO = 50%)

Fluence in J/cm^2	Scanning line overlap LO in % 40	50	60	70	80	90
17	Sa = 1.162μm, (σ = 0.138μm)	Sa = 2.887μm, (σ = 0.527μm)	Sa = 1.689μm, (σ = 0.44μm)	Sa = 5.404μm, (σ = 0.125μm)	Sa = 6.141μm, (σ = 0.09μm)	Sa = 6.896μm, (σ = 0.092μm)
14	Sa = 1.591μm, (σ = 0.084μm)	Sa = 2.929μm, (σ = 0.304μm)	Sa = 2.321μm, (σ = 0.299μm)	Sa = 4.42μm, (σ = 0.106μm)	Sa = 4.755μm, (σ = 0.197μm)	Sa = 6.195μm, (σ = 0.138μm)
11	Sa = 0.86μm, (σ = 0.121μm)	Sa = 1.071μm, (σ = 0.047μm)	Sa = 1.095μm, (σ = 0.047μm)	Sa = 2.875μm, (σ = 0.151μm)	Sa = 3.787μm, (σ = 0.519μm)	Sa = 4.671μm, (σ = 0.123μm)
8	Sa = 0.284μm, (σ = 0.008μm)	Sa = 0.471μm, (σ = 0.058μm)	Sa = 0.253μm, (σ = 0.013μm)	Sa = 0.363μm, (σ = 0.06μm)	Sa = 3.143μm, (σ = 0.376μm)	Sa = 3.552μm, (σ = 0.1μm)
5	Sa = 0.227μm, (σ = 0.004μm)	Sa = 0.244μm, (σ = 0.007μm)	Sa = 0.241μm, (σ = 0.016μm)	Sa = 0.261μm, (σ = 0.007μm)	Sa = 0.926μm, (σ = 0.119μm)	Sa = 2.599μm, (σ = 0.09μm)
3	Sa = 0.175μm, (σ = 0.005μm)	Sa = 0.194μm, (σ = 0.005μm)	Sa = 0.183μm, (σ = 0.007μm)	Sa = 0.251μm, (σ = 0.015μm)	Sa = 0.618μm, (σ = 0.136μm)	Sa = 1.987μm, (σ = 0.043μm)
1.5	Sa = 0.331μm, (σ = 0.005μm)	Sa = 0.16μm, (σ = 0.005μm)	Sa = 0.164μm, (σ = 0.002μm)	Sa = 0.159μm, (σ = 0.003μm)	Sa = 0.255μm, (σ = 0.013μm)	Sa = 0.916μm, (σ = 0.023μm)
0.5	Sa = 0.141μm, (σ = 0.001μm)	Sa = 0.191μm, (σ = 0.001μm)	Sa = 0.142μm, (σ = 0.001μm)	Sa = 0.14μm, (σ = 0.001μm)	Sa = 0.164μm, (σ = 0.003μm)	Sa = 0.178μm, (σ = 0.01μm)

Table S2. Surface area roughness Sa (N = 50, LO = 50%)

Fluence in J/cm^2	Laser pulse overlap PO in % 40	50	60	70	80	90
17	Sa = 0.299μm, (σ = 0.035μm)	Sa = 1.099μm, (σ = 0.217μm)	Sa = 5.13μm, (σ = 0.309μm)	Sa = 7.142μm, (σ = 0.063μm)	Sa = 9.142μm, (σ = 0.217μm)	Sa = 13.463μm, (σ = 0.702μm)
14	Sa = 0.326μm, (σ = 0.041μm)	Sa = 3.113μm, (σ = 0.342μm)	Sa = 5.488μm, (σ = 0.104μm)	Sa = 6.45μm, (σ = 0.069μm)	Sa = 8.478μm, (σ = 0.168μm)	Sa = 13.018μm, (σ = 0.576μm)
11	Sa = 0.289μm, (σ = 0.026μm)	Sa = 0.54μm, (σ = 0.039μm)	Sa = 3.188μm, (σ = 0.163μm)	Sa = 4.507μm, (σ = 0.06μm)	Sa = 5.461μm, (σ = 0.171μm)	Sa = 8.321μm, (σ = 0.431μm)
8	Sa = 0.286μm, (σ = 0.052μm)	Sa = 0.301μm, (σ = 0.005μm)	Sa = 1.79μm, (σ = 0.197μm)	Sa = 3.766μm, (σ = 0.052μm)	Sa = 5.166μm, (σ = 0.16μm)	Sa = 7.686μm, (σ = 0.475μm)
5	Sa = 0.192μm, (σ = 0.035μm)	Sa = 0.191μm, (σ = 0.007μm)	Sa = 0.296μm, (σ = 0.031μm)	Sa = 2.937μm, (σ = 0.069μm)	Sa = 3.517μm, (σ = 0.238μm)	Sa = 7.264μm, (σ = 0.164μm)
3	Sa = 0.191μm, (σ = 0.012μm)	Sa = 0.158μm, (σ = 0.004μm)	Sa = 0.198μm, (σ = 0.007μm)	Sa = 0.625μm, (σ = 0.058μm)	Sa = 2.2μm, (σ = 0.11μm)	Sa = 3.327μm, (σ = 0.61μm)
1.5	Sa = 0.207μm, (σ = 0.004μm)	Sa = 0.171μm, (σ = 0.005μm)	Sa = 0.161μm, (σ = 0.004μm)	Sa = 0.223μm, (σ = 0.016μm)	Sa = 0.597μm, (σ = 0.017μm)	Sa = 1.299μm, (σ = 0.139μm)
0.5	Sa = 0.125μm, (σ = 0.058μm)	Sa = 0.201μm, (σ = 0.001μm)	Sa = 0.224μm, (σ = 0.002μm)	Sa = 0.266μm, (σ = 0.002μm)	Sa = 0.294μm, (σ = 0.002μm)	Sa = 0.392μm, (σ = 0.004μm)
